# Ubenimex attenuates acquired sorafenib resistance in renal cell carcinoma by inhibiting Akt signaling in a lipophagy associated mechanism

**DOI:** 10.18632/oncotarget.13003

**Published:** 2016-11-01

**Authors:** Shuai Liu, Mingwei Gao, Xiaoqing Wang, Sentai Ding, Jiaju Lv, Dexuan Gao, Zhiyang Wang, Zhihong Niu

**Affiliations:** ^1^ Department of Urology, Shandong Provincial Hospital Affiliated to Shandong University, Jinan, 250021, China

**Keywords:** renal cell carcinoma, sorafenib resistance, lipophagy, Akt pathway, ubenimex

## Abstract

Sorafenib is used as first line treatment of renal cell carcinoma (RCC) due to the poor sensitivity to radiotherapy and chemotherapy of this malignancy; however, acquired resistance limits the application of sorafenib and its analogues. In this study, we explored a new strategy to overcome acquired resistance to sorafenib. The RCC cell lines 786-O and ACHN were cultured in presence of increasing concentrations of sorafenib to generate sorafenib-resistant cell lines, 786-O-R and ACHN-R. Interestingly, treatment with ubenimex (0.25 mg/ml) and 3-MA (2 mM) restored the sensitivity of resistant cell lines to sorafenib, indicating the involvement of autophagy in acquired resistance. High levels of autophagy flux were observed in resistant cells, and the opposite effects of ubenimex and 3-MA suggested a complex role for autophagy. While 3-MA abolished protection in sorafenib-resistant cells, ubenimex induced uncontrolled autophagy and autophagic cell death. Lipophagy, characterized by a lipid droplet cargo, was observed in RCC tissues and cells. In sorafenib-resistant cells, ubenimex inhibited the Akt signaling pathway that regulates autophagy. In summary, lipophagy participates in sorafenib-resistance of RCC, which could be reversed by interventions targeting the Akt pathway.

## INTRODUCTION

Renal cell carcinoma (RCC), characterized by high malignancy and poor prognosis, is one of the most common malignant tumors of the adult urinary system. Approximately 30% of RCC patients are diagnosed with metastatic or advanced carcinoma at first visit. Furthermore, 20 to 40% of individuals with local carcinoma who undergo surgical resection subsequently develop metastatic disease [1]. Following the pivotal phase 3 TARGET (Treatment Approaches in Renal Cancer Global Evaluation Trial) clinical trial, sorafenib has become the first line targeted therapy for metastatic RCC. Sorafenib also shows efficacy in patients with no response to previous therapy [2]. However, several factors, including the low proportion of patients achieving complete or partial response and resistance associated with a sustained use, clearly demonstrate sorafenib inadequacies. Therefore, a novel adjuvant is required to enhance sensitivity to sorafenib and reverse resistance.

Autophagy is a process of self-digestion, whereby cytoplasmic components are engulfed and destroyed by double-membraned autophagosomes. It normally occurs at low levels in normal cells and helps cells eliminate aberrantly folded proteins, repairing the damage caused by stressors such as temporary nutrient withdrawal, low O_2_ concentrations, and metabolic waste accumulation. Uncontrolled autophagy results in cell death. Studies have shown that altered autophagy proteins promote cell death, and autophagy can be required for apoptosis and other types of cell death [3]. The recent finding that lipids can be selectively degraded by the lysosomal pathway of macroautophagy, through a process termed lipophagy, has provided new highlights regarding how lipid metabolism regulates cellular physiology and pathophysiology. Many new functions for autophagic lipid metabolism have now been defined in diverse cellular processes, ranging from transdifferentiation to resistance to cell death.

In addition to amino acids, increased levels of free fatty acids (FFAs) and sugars are found in tissues undergoing high levels autophagy. In this case, autophagy is associated with lipid metabolism, a process known as lipophagy. Stored lipid droplets (LDs) and FFAs are taken up as an alternative route of lipid metabolism to provide energy. For example, latest research showed lipophagy mediates LD degradation and lipolysis in androgen-sensitive PCa cells during androgen deprivation, which increases survival in PCa cells during hormone therapy, indicating that lipophagy plays an important role in drug resistance [4].

The Akt signaling pathway is activated in many carcinoma cells. Akt expression is regulated by its downstream effector, the mammalian target of rapamycin (mTOR), which suppresses the formation of the UVRAG, Vps15, Vps34, and Beclin-1 complex. Beclin-1 plays a key role in autophagy, and Akt directly downregulates the anti-apoptotic protein Bad.

This study aimed to assess the roles of autophagy and lipid metabolism (lipophagy) in sensitivity and resistance to sorafenib, providing evidence for identifying interventions that reverse sorafenib-resistance.

## RESULTS

### Sorafenib-resistant RCC cells are refractory to sorafenib

**Figure 1:**
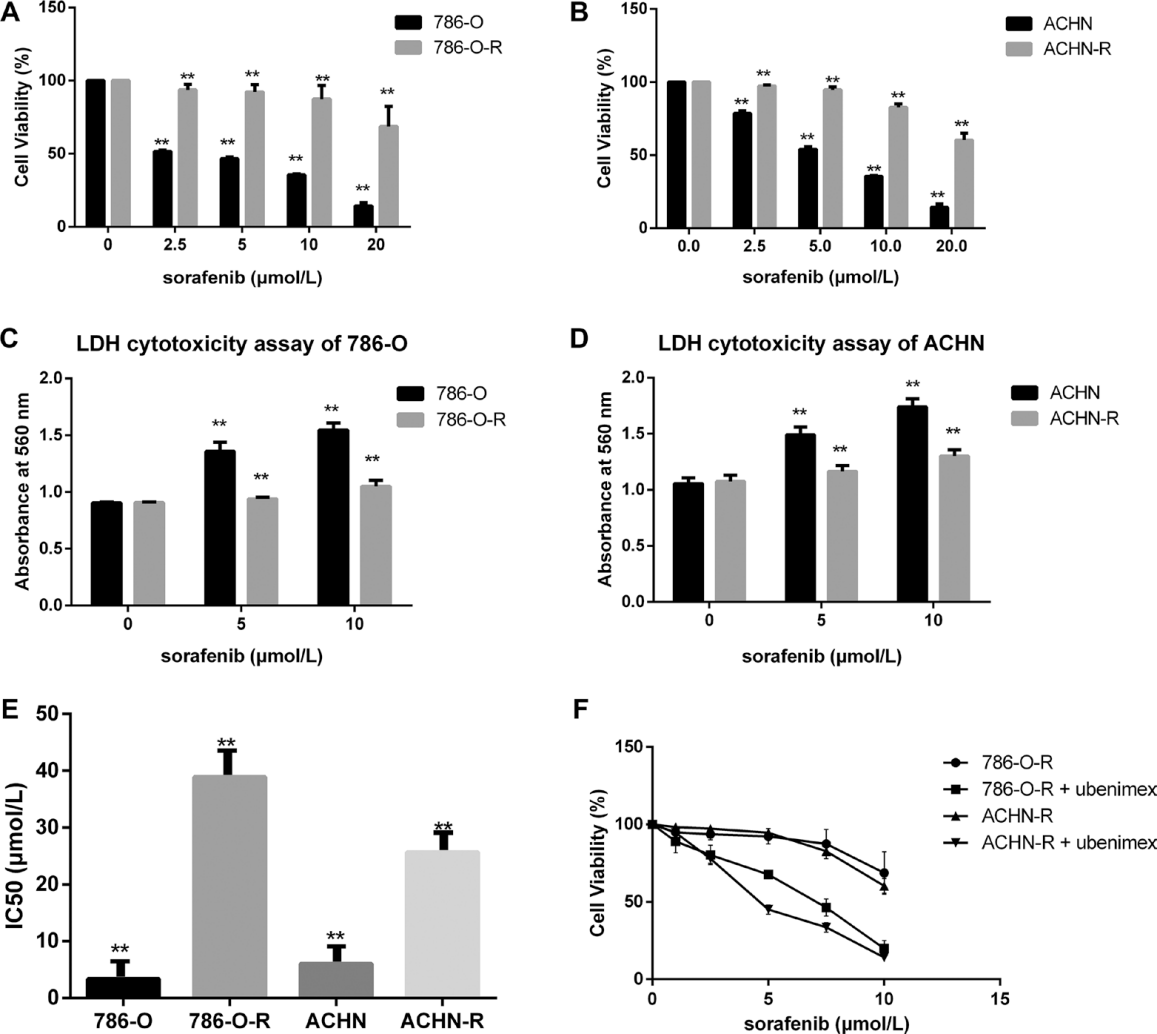
Sorafenib resistant cells show lower susceptibility (**A**, **B**) Sorafenib resistant cells had higher cell viability. (**C**, **D**) To estimate the extent of cell death, LDH cytotoxicity assay was used, with absorbance measured at 560 nm. In the first sorafenib treatment, cell lines showed overt growth inhibition and increased cell death. (**F**) Ubenimex inhibited resistant cells. Data represent three independent experiments. **P < 0.001 versus sorafenib sensitive cells.(**E**):Sorafenib-resistant cells showed higher IC_50_ values for sorafenib compared with parental cells (*P < 0.05 vs parental cells).

**Figure 2:**
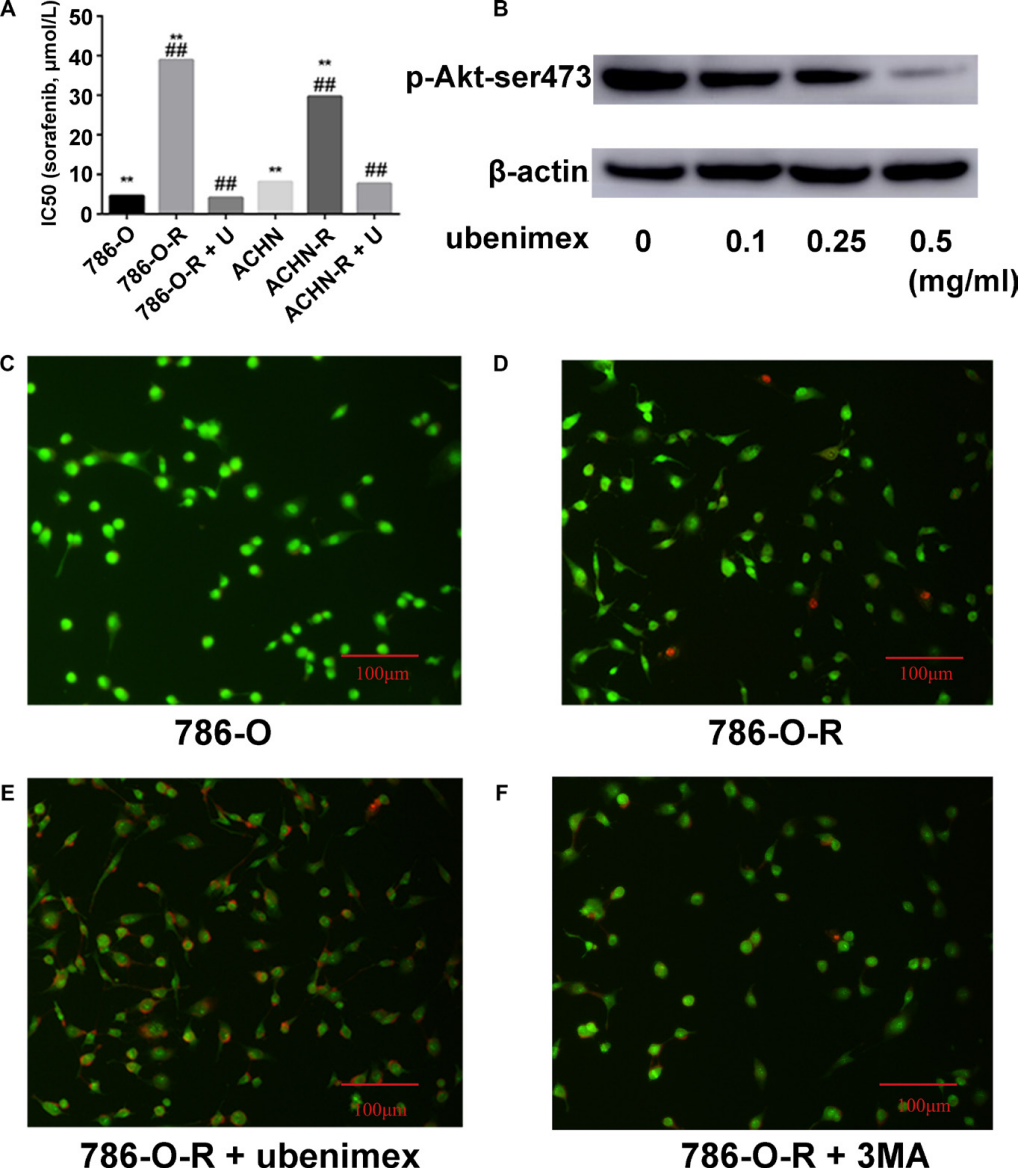
(**A**) Resistant cells display higher IC^50^ for sorafenib. After treatment with ubenimex, both 786-O-R and ACHN-R displayed low IC^50^ values for sorafenib. (**B**) Ubenimex dose dependently inhibited p-Akt-ser473 expression. Without treatment, 786-O (**C**) and 786-O-R (**D**) cells showed very low levels of apoptosis as assessed by AO/EB staining. (**E**) Ubenimex enhanced apoptosis in sorafenib resistant cells. (**F**) Inhibition of autophagy by 3MA resulted in enhanced apoptosis in sorafenib resistant cells, but no more than ubenimex. Data represent three independent experiments. **P < 0.001 versus sorafenib resistant cells; ^##^*P* < 0.001 versus sorafenib resistant cells treated with ubenimex. Bars, 100 μm.

**Figure 3:**
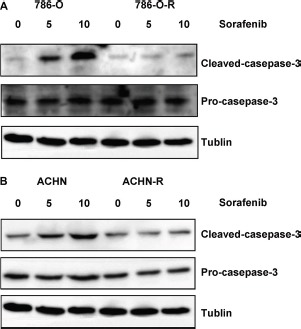
Western blot assessing cleaved caspase-3 and pro-caspase-3 showed that both sorafenib-resistant and parental cells exhibited similar apoptosis rates at baseline (no sorafenib treatment control); however, after treatment with sorafenib, sorafenib-resistant cells showed lower cleaved caspase-3 levels compared with parental cells

**Figure 4:**
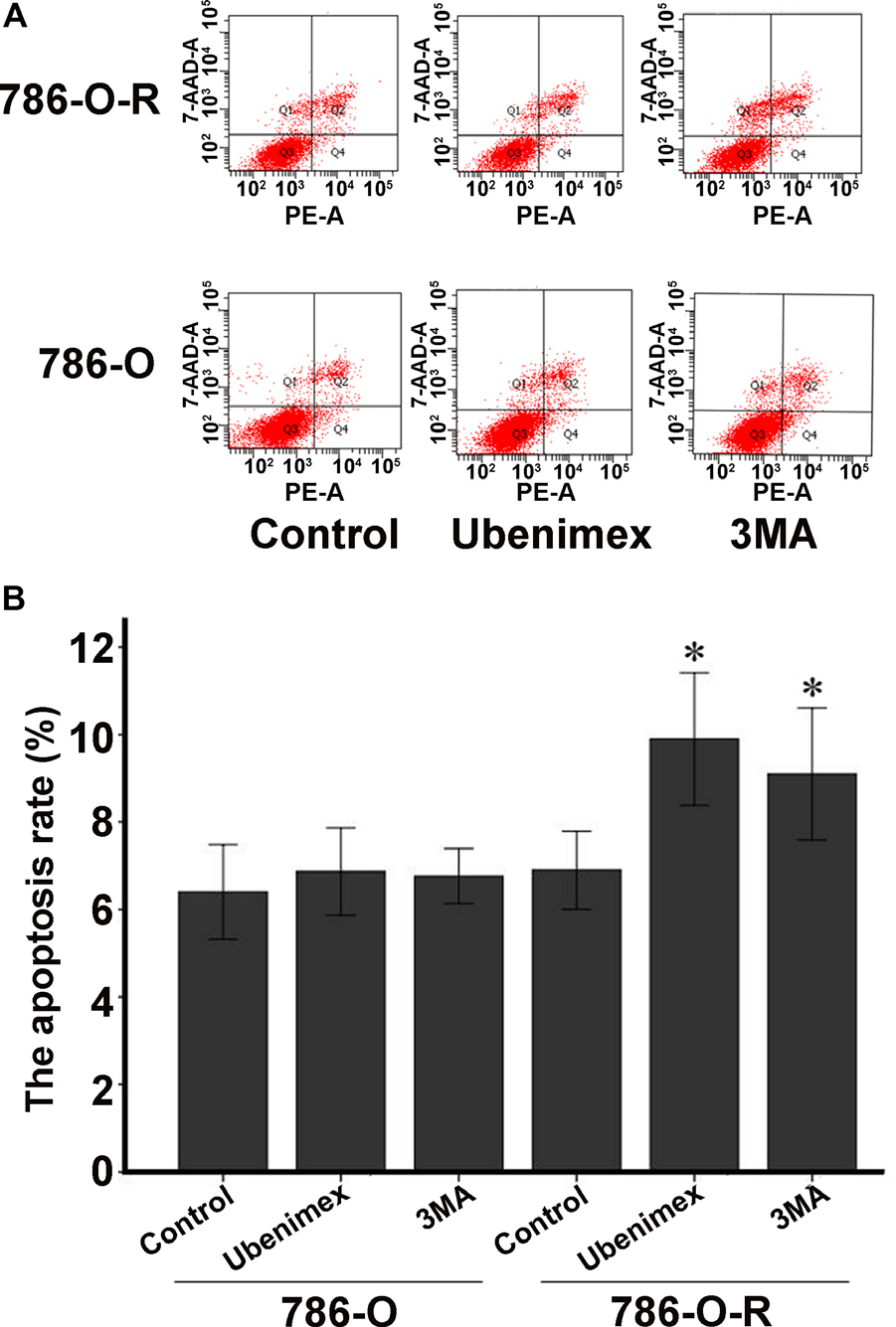
Annexin V-PI staining assessing 786-O and 786-O-R apoptosis rates after ubenimex treatment Ubenimex induced apoptosis in 786-O-R cells, an effect attenuated by the autophagy inhibitor 3MA, indicating a cross-talk between autophagy and apoptosis. (**A**) Flow-cytograms; (**B**) quantitation of a (*P < 0.05).

After incubation with 8 μmol/L sorafenib for 10 passages, the RCC cell lines 786-O-R and ACHN-R became refractory to growth inhibition and cell death induced by sorafenib. Viability of 786-O-R and ACHN-R cells exposed to different doses of sorafenib was higher than that of parental 786-O and ACHN cells (*P* = 0.011, Figure 1A–1B). In this study, half maximal inhibitory concentration (IC_50_) values were used to evaluate sorafenib efficacy. Compared with parental cell lines, sorafenib-resistant cells showed higher IC_50_ values (*P* = 0.016, Figure 1E). Cell death was evaluated by determining the levels of lactate dehydrogenase (LDH) released. Resistant cells showed reduced death compared with respective parental cells at the same sorafenib dose (*P* = 0.002, Figure 1C–1D), while both resistant and parental cells exhibited similar rates of apoptosis at baseline (no sorafenib treatment control) by AO-EB staining (Figure 2C, 2D). This was also verified by Western blot (Figure 3) and Annexin V-PI staining (Figure 4). Studies have indicated that sorafenib enhances the rate of apoptosis [5–8]. As a member of the anti-apoptotic Bcl family, the Bcl-2 protein was selected to evaluate cell apoptosis. The anti-apoptotic function Bcl-2 was inhibited by sorafenib in a dose-dependent manner. Western blot analysis of cleaved-caspase-3 and pro-caspase-3, which play a key role in apoptosis, revealed the ability of sorafenib to induce the expression of these proteins, an effect also attenuated in sorafenib-resistant cells (Figure 3).

### Ubenimex enhances sorafenib efficiency and reverses resistance

Treatment with ubenimex (0.25 mg/ml), an aminopeptidase N (APN) or CD13 inhibitor, enhanced sorafenib-resistance inhibition as well as death ratios in RCC cells (Figure 2E). Ubenimex reduced the viability of resistant cells, and reversed resistance (*P* = 0.003, Figure 1F). IC_50_ values of sorafenib-resistant cells were higher than those obtained for sorafenib-sensitive cells (*P* = 0.017); this effect was reversed by treatment with ubenimex (*P* = 0.008, Figure 2A).

### Autophagy is involved in sorafenib-resistance

**Figure 5:**
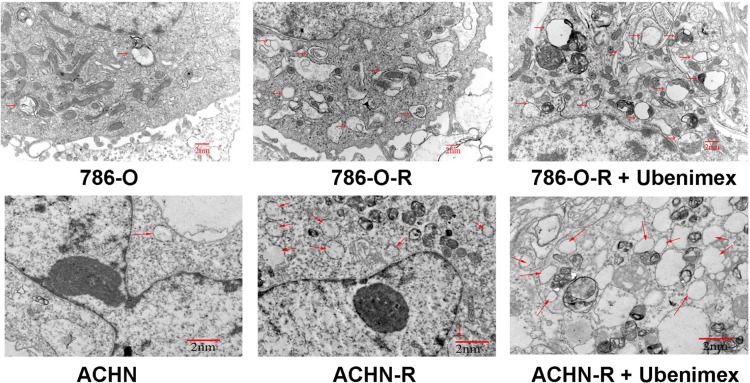
Electron microscopy of ACHN, ACHN-R, and ACHN-R+ ubenimex →, autophagosome. Bars, 2 μm.

**Figure 6:**
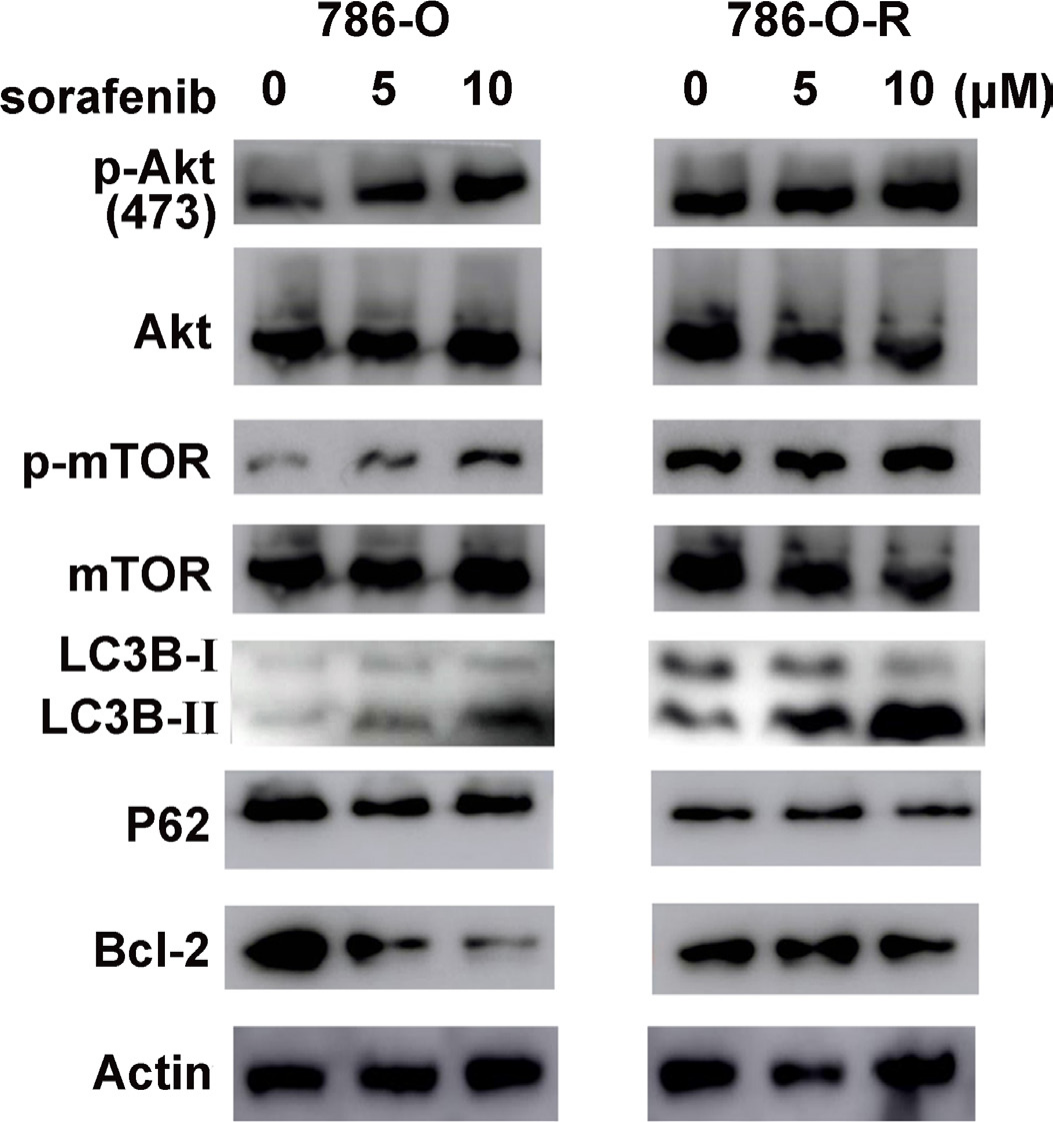
The Akt pathway is activated in sorafenib resistant cells p-Akt-ser473 levels increased dose-dependently, but total Akt expression showed no difference. As a downstream effector of p-Akt-ser473, p-mTor-ser2481 showed similar changes. Sorafenib resistant cells had higher expression of Akt pathway effectors and increased autophagy levels (LC3B and P62 amounts).

**Figure 7:**
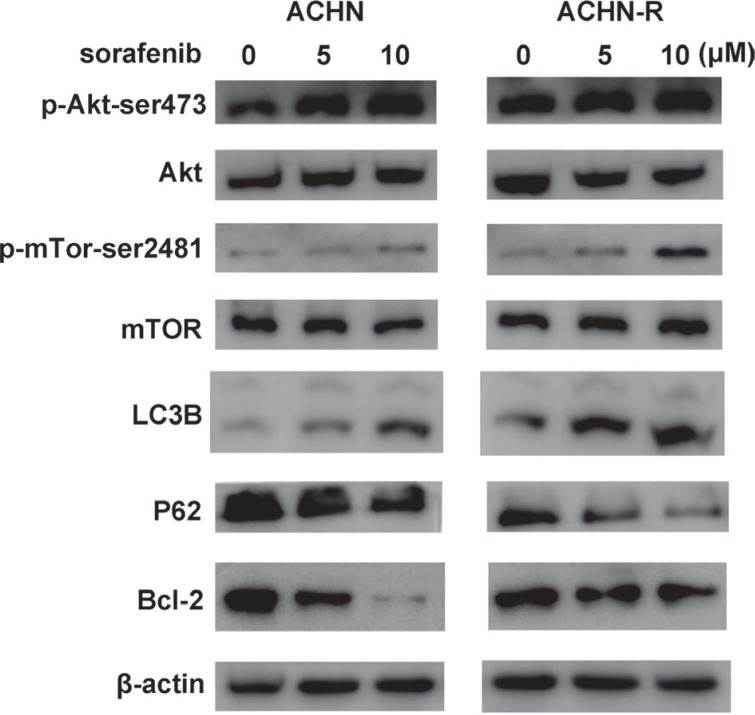
Similar changes in Western blot were found for another RCC cell line, ACHN

Autophagy is believed to play a protective role in tumor cells; thus, we hypothesized that it participates in the mechanism underlying sorafenib-resistance. Sorafenib causes a stress response in cells to overcome autophagy. 3-Methyladenine (3-MA) is often used to suppress autophagy by inhibiting phosphoinositide 3-kinase (PI3K) III and Vps34. 3-MA treatment resulted in reduced sorafenib-resistance (Figure 2F), indicating that protection against autophagy contributed to this phenomenon. A high count of autophagosomes was observed in ACHN-R (Figure 5). Western blot confirmed the high levels of autophagy in 786-O-R and ACHN-R cells (Figure 6, Figure 7). Autophagy increased with sorafenib dose, although the amounts in untreated resistant cells remained high. Annexin V-PI staining demonstrated that Ubenimex induced apoptosis in 786-O-R cells; this effect was attenuated by the autophagy inhibitor 3MA, indicating a cross-talk between autophagy and apoptosis (Figure 4).

### Lipophagy changes in resistant cells

Lipophagy is characterized by a LD cargo in autophagosomes. Tumor cells require the high energy levels, and lipid oxidation provides more energy per unit mass than that of carbohydrates. High lipophagy levels and increased storage of LDs have been observed in some arcinomas. Therefore, we hypothesized that lipophagy may be increased in sorafenib-resistant cells.

**Figure 8:**
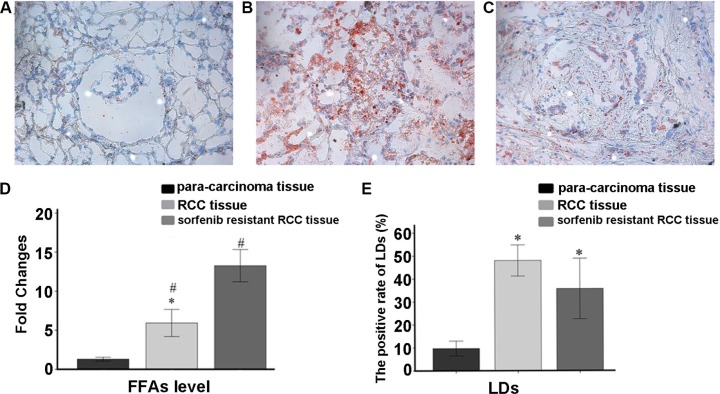
Oil Red O staining data (**A**) Para-carcinoma; (**B**) RCC tissue; (**C**) sorafenib resistant RCC tissues. (**D**) Average fold changes of each group in FFA levels measured by ELISA. Sorafenib resistant RCC tissues had higher lipophagy and FFA amounts. Data represent three independent experiments. *P < 0.01 versus fold changes of FFA amounts in RCC tissue specimens; ^#^*P* < 0.01 versus sorafenib resistant RCC tissue samples (**E**) The positive rate of LDs in slides.*P < 0.01 versus positive rate of LDs in para-carcinoma tissue specimens.

**Figure 9:**
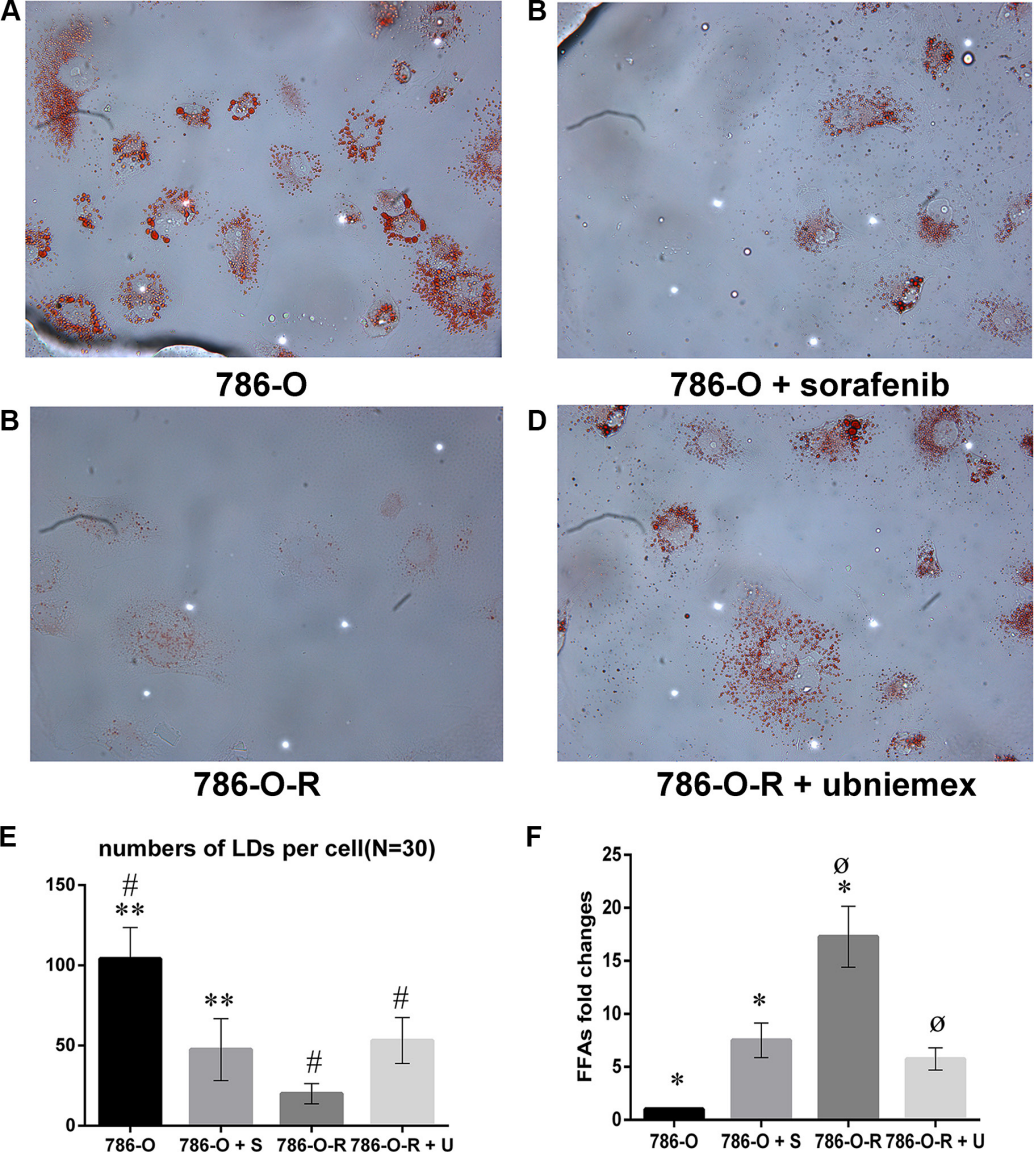
Storage of lipid droplets (LDs) was higher after treatment with sorafenib (A, B) In sorafenib resistant cells, treatment with sorafenib did not overtly change LD levels (**C**, **D**). Data represent three independent experiments. *P < 0.01 versus 786-O dealing with sorafenib at 5 μmol/L; ^#,ø^*P* < 0.01 versus 786-O-R + ubenimex. Data represent three independent experiments. *P < 0.01 versus 786-O dealing with sorafenib at 5 μmol/L; ^#,ø^*P* < 0.01 versus 786-O-R + ubenimex (**E**, **F**).

Oil red O staining of frozen RCC tissue sections from sorafenib-resistant patients showed numerous LDs (*P* = 0.007, Figure 8). *In vitro* experiments also revealed high free fatty acid (FFA) levels in sorafenib-resistant patients (*P* = 0.002; Figure 8 and Figure 9).

Compared with 786-O cells, 786-O-R cells had a reduced level of LD storage, indicating a more significant consumption in sorafenib-resistant cells. As a product of lipophagy, the high FFA levels in 786-O-R cells indicated a high rate of lipophagy.

### Activation of the Akt signaling pathway in sorafenib-resistant cells

Activation of the Akt signaling pathway is found in many types of carcinomas. Sorafenib treatment resulted in a dose-dependent increase of p-Akt-ser473 expression in sorafenib-resistant cells, although no obvious change was observed in total Akt levels (Figure 6, Figure 7). Furthermore, p-Akt-ser473 amounts were maintained at a higher level in sorafenib-treated resistant cells compared with sensitive cells treated at the same dose (Figure 6, Figure 7).

### Ubenimex attenuates resistance by enhancing autophagy and lipophagy and suppressing Akt signaling pathway

**Figure 10:**
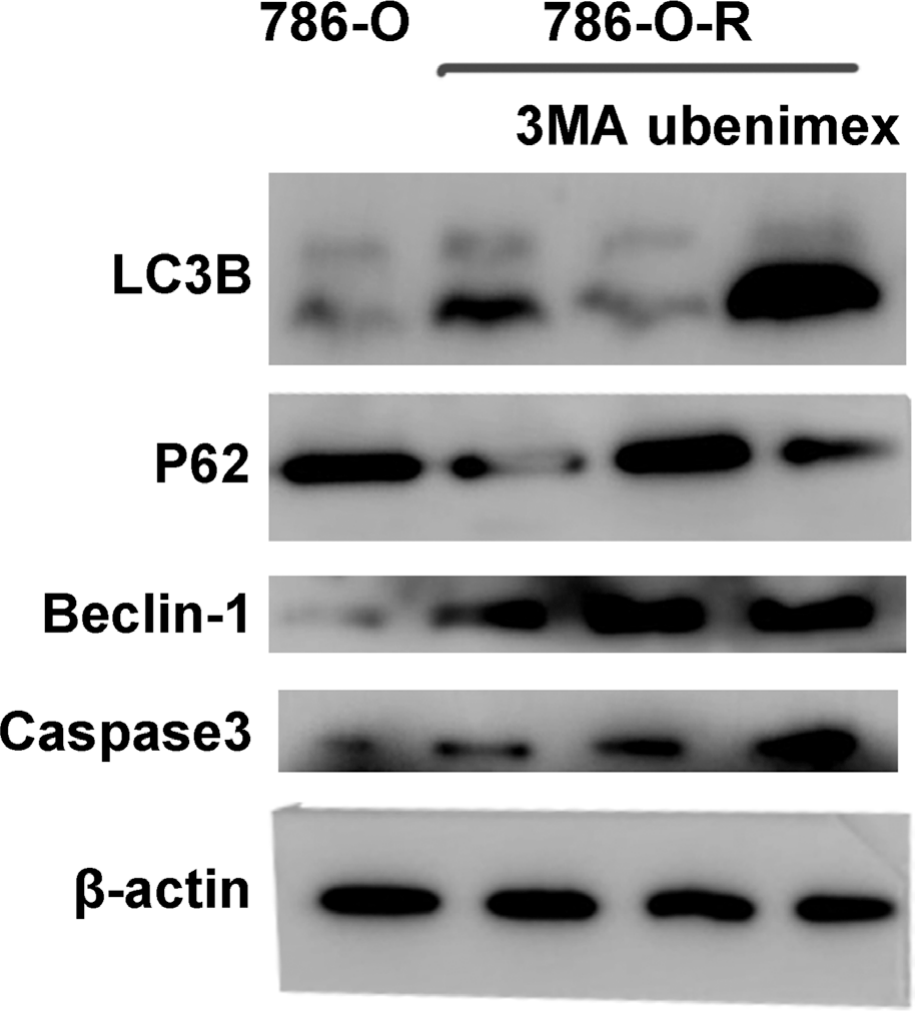
786-O, 786-O-R, 786-O-R + 3MA, 786-O-R + ubenimex were selected for Western blot 786-O-R cells showed a high level of autophagy.

The overtly higher autophagy levels demonstrated by Western blot indicated that ubenimex and 3-MA reverse acquired sorafenib-resistance via different mechanisms (Figure 10). Excessive uncontrolled autophagy causes cell structure destruction, and ultimately induces autophagic cell death. Ubenimex clearly suppressed p-Akt-ser473 and protein expression, in a dose-dependent manner (Figure 2B).

### Ubenimex reverses resistance to sorafenib *in vivo*

**Figure 11:**
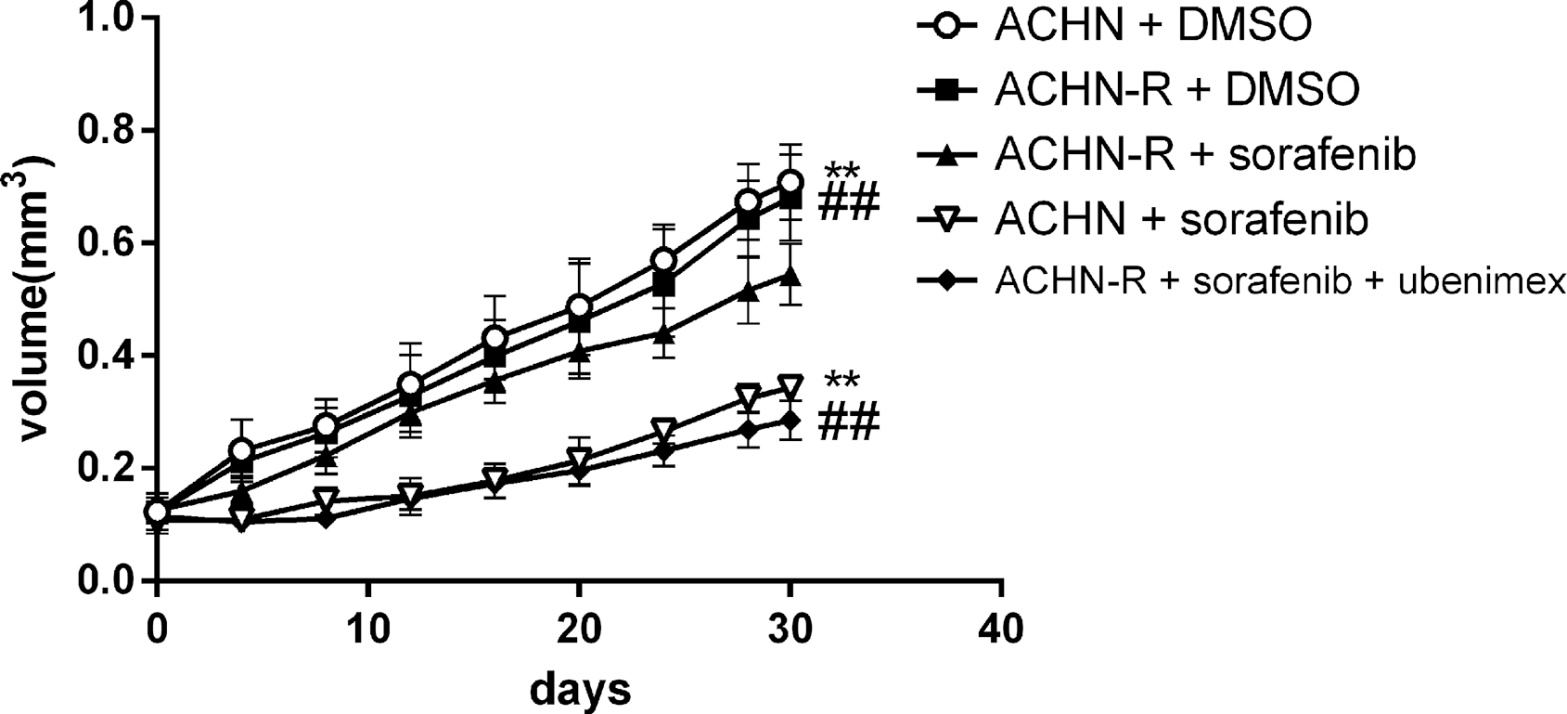
Subcutaneous injection of 5*10^6^ ACHN cells per nude mouse was carried out At 2 weeks (set as day 0), treatment was initiated and tumor lengths and widths were measured every 4 days. Tumor volume was derived as length × width ^2^/2. Sorafenib at 30 mg/kg daily inhibited tumor growth; however, in sorafenib resistant xenografts, no obvious difference was found between the vehicle and sorafenib groups. Interestingly, treatment with ubenimex at 20 mg/kg daily reversed the resistance of the xenografts.

**Figure 12:**
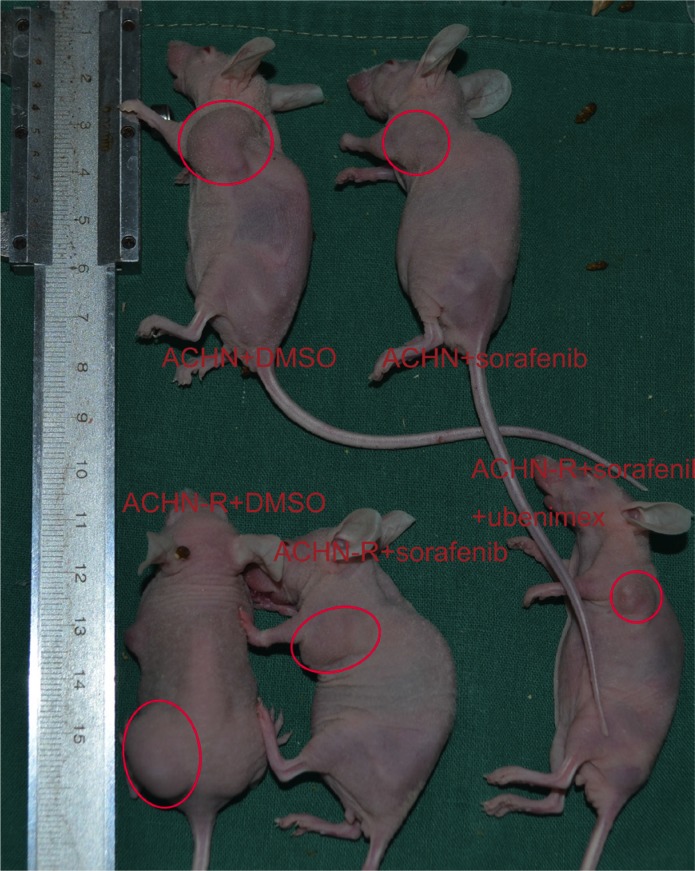
Representative data for each mouse group

*In vivo* experiments in mice showed that tumor volumes for resistant cells increased faster than those of the parental cell lines (*P* = 0.004, Figure 11). In ACHN-R xenografts treated with sorafenib, tumor inhibition was observed, although this effect was less pronounced than that observed in ACHN cell xenografted mice treated with sorafenib. Interestingly, ubenimex in combination with sorafenib showed far greater inhibition of ACHN-R tumor growth, with no apparent difference compared with the ACHN + sorafenib group (Figure 11, Figure 12).

## DISCUSSION

An improved understanding of the mechanisms underlying acquired sorafenib-resistance is urgently required to design strategies to overcome this limitation. The PI3K/Akt pathway is frequently activated in many cancers, and previous studies have revealed a convergence of PI3K/Akt/mTOR activation through a variety of mechanisms in clear RCC [9]. Growth factor stimulation activates the PI3K pathway. As a representative of tyrosine kinase inhibitors, the main mechanism underlying tumor angiogenesis suppression by sorafenib involves inhibition of vascular endothelial growth factor (VEGF), vascular Endothelial Growth Factor Receptor (VEGFR), and platelet derived growth factor (PDGF).

**Figure 13:**
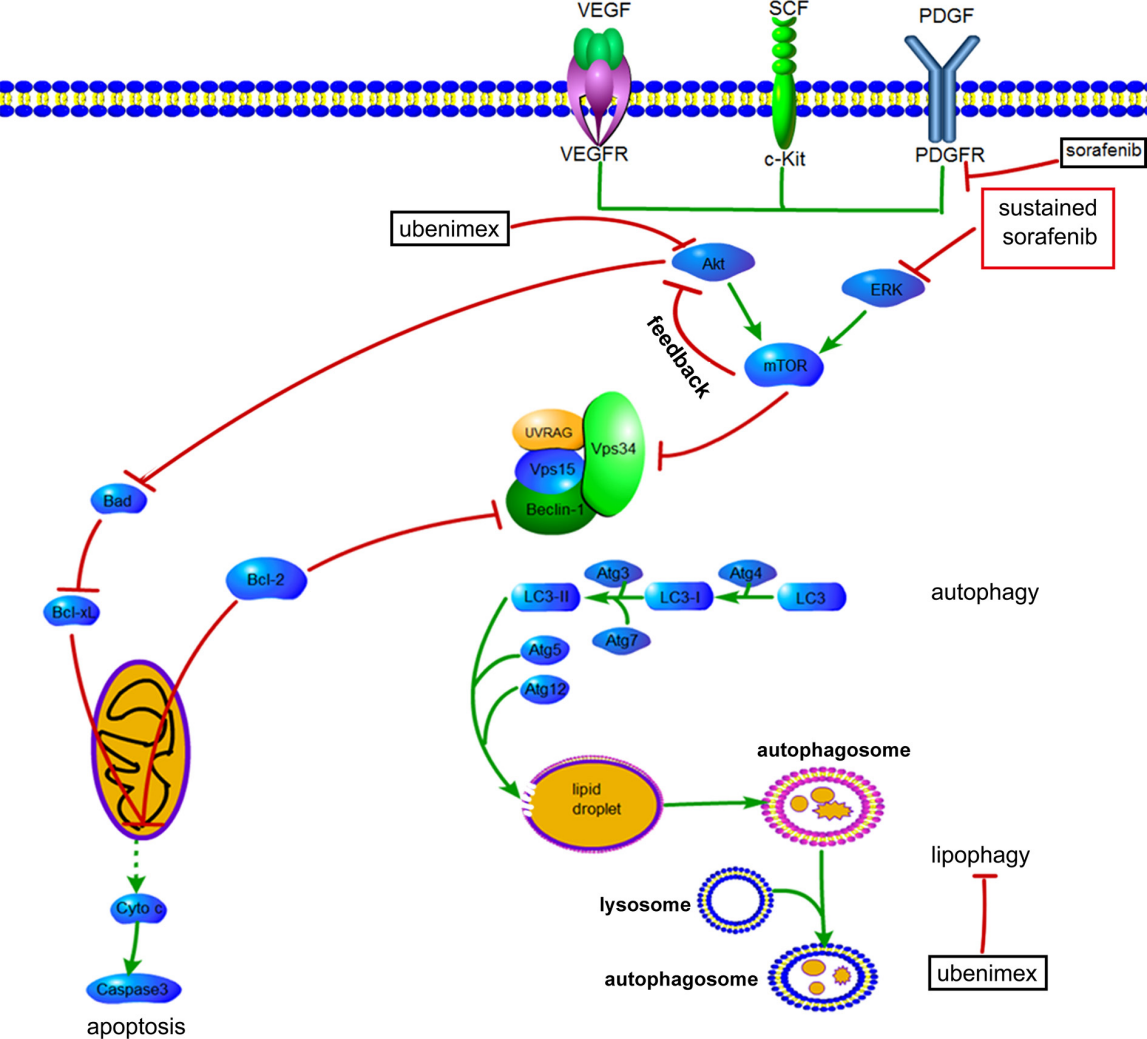
Proposed mechanisms by which AKT activation contributes to acquired resistance to sorafenib by regulating Akt and autophagy →, positive regulation or activation; ⊥, negative regulation or blockade.

Inhibition of mTOR regulates the upstream signaling molecule Akt, by a feedback mechanism (Figure 13). Chronic exposure of sorafenib-resistant RCC cells to sorafenib inhibits ERK activation of [10], sequentially reduces mTOR independent of the PI3K/Akt pathway [11], and activates Akt via a feedback loop. Downregulation of the Akt kinase Akt1 enhances the sensitivity of ACHN cells to sorafenib both *in vitro* and *in vivo* by regulating apoptosis-related molecules [12]. Zhai et al. reported that sustained exposure to sorafenib activates Akt in hepatic carcinoma cells via the mTOR feedback loop. Furthermore, inhibiting Akt reverses acquired sorafenib-resistance by converting autophagy from a cytoprotective role to a death-promoting one in sorafenib-resistant hepatocellular carcinoma cells [13]. Meanwhile, mTOR inhibits the formation of the UVRAG, Beclin-1, Vps15, and Vps34 complex, which is essential for the autophagy process [14]. Rapamycin, which inhibits mTORC1, is widely used to induce autophagy. As shown in Figure 4 and Figure 5, sorafenib induced Akt upregulation and autophagy, in sharp contrast with previous studies suggesting that Akt pathway upregulation inhibits autophagy; this discrepancy may be explained by many factors. Stress conditions, such as low oxygen, lack of nutrition, and medication, could activate protective autophagy to enhance cell survival. Bcl-2 family members are anti-apoptotic proteins which bind to Beclin-1 to inhibit autophagy by preventing complex formation between Beclin-1 and class III PI3K. Sorafenib enhances RCC apoptosis, and Bcl-2 downregulation promotes autophagy.

The impacts of PI3K signaling on autophagy and apoptosis implicate this pathway in the anti-tumor mechanism of sorafenib. It was suggested that tumor cells undergo protective autophagy to overcome the anti-tumor effects of medications [15]. Meanwhile, Fischer et al. demonstrated higher autophagy flux in sorafenib-resistant cell lines compared with sensitive cells [16]. Furthermore, while autophagy promotes sorafenib-resistance in hepatocellular carcinoma cells [17], inhibition of autophagy enhances the anti-tumor effects of sorafenib [18]. This is supported by our findings that 3-MA reversed sorafenib-resistance by inhibiting protective autophagy. Previous studies indicated that at very low concentrations, sorafenib works by inducing autophagic cell death instead of apoptosis. With increasing concentration and treatment time, the anti-tumor effects of sorafenib depend mainly on apoptosis, and the role of autophagy changes from an anti-tumor type to a tumor promoting one [19]. On the other hand, inducing autophagic cell death could be an effective strategy to overcome sorafenib-resistance [13].

Although stress conditions, such as starvation and certain types of medication, could enhance autophagy, the extra energy provided by enhanced autophagy cannot satisfy the high requirements for energy in tumor cells. Autophagy is classified according to its cargo. The LDs accumulated in lipophagy may represent another response of tumor cells to address the energy crisis. Analysis of autophagosome components in cells revealed the presence of lipid materials and LD structural proteins inside the vesicles [20]. Autophagy enhanced lysosomes do not fuse directly with LDs, but with LD-contained autophagosomes. LC3 is recruited to the LDs, where it initiates cargo formation via ATG7-dependent conjugation in hepatocytes [20]. Although no studies of lipophagy in renal carcinoma have been reported, increased autophagy in resistant RCC raises the possibility that the increased lipophagy level in resistant RCC is an important mechanism of resistance. The high LD levels observed in this study indicated the involvement of lipophagy in providing the energy required for RCC tumorigenesis.

Increased FFA generation by increased lipophagy has been demonstrated in neurons, the muscle and pancreas, mammary epithelial cells, liver-derived cells, and colon cancer cells [21–25]. It was shown that accumulation of FFAs, especially saturated FFA palmitate, causes mitochondrial dysfunction and reactive oxidative species generation, inducing disruption of cellular homeostasis and cell death [26]. Significantly increased FFA levels have been reported in hepatic carcinoma patients, with effects dependent upon the mTOR/NF-κB pathway [27]. Munkarah et al. demonstrated that FFAs promote high grade serous ovarian carcinoma in a mouse model, and targeting FFA activated G-protein coupled receptors inhibits tumor growth [28]. However, Zhang et al. suggested that impairment of a unique set of FFAs inhibits pancreatic cancer development and progression [29]. No obvious differences in LD storage were observed between sorafenib-sensitive and sorafenib-resistant cells; however, the higher FFA amounts observed in resistant cells suggest a role for FFA metabolism in the development of sorafenib-resistance.

Changes in APN (CD13) expression in tumor tissues and serum are associated with decreased survival rates and poor prognoses in lung, colorectal, pancreatic and renal carcinomas [30]. Our previous study showed higher levels of APN (CD13) in carcinoma tissues compared with para-carcinoma ones. As an APN inhibitor, ubenimex suppresses cell proliferation, migration and invasion in RCC by autophagy. Ubenimex suppresses Akt expression and enhances autophagy (Figure 2A, 2B). Uncontrolled autophagy threatens cell survival by inducing autophagic cell death. Furthermore, ubenimex enhanced apoptosis in 786-O-R cells (Figure 2) and decreased FFA levels to reverse sorafenib-resistance.

In conclusion, high levels of autophagy and lipid metabolism (lipophagy) play a role in the protective mechanisms of cancer cells. Ubenimex downregulates p-Akt-ser473 and induces autophagic cell death to reverse sorafenib-resistance. Thus, ubenimex showed an expected effect as an adjuvant.

## MATERIALS AND METHODS

### Cell culture

The RCC cell lines 786-O and ACHN were purchased from American type culture collection (ATCC). 786-O was cultured in RPMI-1640 (Life Technologies Inc., Gaithersburg, MD, USA) supplemented with 10% FBS, penicillin, and streptomycin at 37°C in a humid environment containing 5% CO_2_. ACHN cells were maintained in the same conditions in MEM (HyClone Biotechnology, Carlsbad, CA, USA) instead. IC_50_ values were measured by incubating cells with a series of sorafenib concentrations in 96-well plates, with a procedure similar to the WST-8 assay described below. Then, the cells were incubated with a concentration just below their respective IC_50_. This concentration was increased gradually by 0.5 μmol/L every two passages. When sorafenib amounts reached 8 μmol/L, the cells were further incubated for > 10 passages before use in subsequent experiments.

### WST-8 cell proliferation assay

Cells were cultured to 70% confluency, and seeded in 96-well plates at a density of 3,000 cells/well in 90 μl complete medium supplemented with stepwise escalation of drug concentrations. After 24 h of incubation, 10 μl of the WST-8 solution (WST-8 cell proliferation and cytotoxicity assay kit; Dojindo, Kumamoto, Japan) was added into each well, and incubated for an additional 1h at 37°C. Absorbance was determined on a microplate reader (EL340 Bio-Tek Instruments, Hopkinton, MA, USA) at 450 nm.

### LDH release assay

Released LDH amounts were assessed as an indicator of the extent of cell death, including all types of death. 200 μl cell suspension was seeded in each well of a 96-well plate (5 × 10^3^ cells/well). Ubenimex (0.25 mg/ml) or 3-MA (2 mM) was added for 24 h. After centrifugation at 400 g for 5 min, 120 μl of the resulting supernatant from each well was transferred into a new plate. The plates were incubated at room temperature for 30 min in the dark, and absorbance was spectrophotometrically measured at 560 nm.

### Dual AO/EB fluorescent staining

Cells were seeded into 6-well plates at a final density of 10^4^/ml, and treated with stepwise escalation of ubenimex and sorafenib concentrations. After 6 h, the cells were washed with PBS twice. Dual fluorescent staining solution (1 μl) mixed with 100 μg/ml AO and 100 μg/ml EB (AO/EB, Sigma, St. Louis, MO) was added to each well, and covered with a coverslip. The morphology of apoptotic cells was observed under a fluorescence microscope (OLYMPUS, Japan). Dual acridine orange/ethidium bromide (AO/EB) staining was repeated at least 3 times.

### Western blot

Parental and respective sorafenib resistant cells were seeded into 6-well plates and treated with different drug concentrations for 24 h. Before lysis, cells were washed once with ice-cold PBS. Proteins were extracted with the RIPA buffer containing 1% of PMSF and phosphatase inhibitor cocktails. Cells were collected into Eppendorf tubes and incubated on ice with the lysis buffer mixture for 30 minutes. The lysates were centrifuged at 12,000 rpm and 4°C for 30 minutes, and the resulting supernatants were used for analysis. Protein concentrations were assessed with the Bradford protein method, using the BCA protein assay kit (Solarbio, Beijing, China). Equal protein amounts (40 μg) were electrophoresed on pre-cast Bis-Tris polyacrylamide gels (8% and 12%) and transferred onto PVDF membranes. The membranes were blotted with rabbit anti-LC3B (1:1,000; Sigma), anti-Caspase3 (1:1,000, CST; Danvers, MA, USA), anti-Akt (1:1,000, CST; Danvers, MA, USA), anti-pAkt-ser473 (1:1,000, CST; Danvers, MA, USA), anti-mTOR (1:1,000, CST; Danvers, MA, USA), anti-pmTOR-ser2481 (1:1,000, CST; Danvers, MA, USA), anti-Bcl-2 (1:1,000, Abcam, UK) and anti-β-actin (1:1,000, BOSTER, China) primary antibodies, followed by incubation with horseradish peroxidase (HRP)-conjugated secondary antibodies (1:5,000; ZB2306 and ZB2301; ZsBio, Beijing, China). Immunoblots were developed by enhanced chemiluminescence (LAS4000).

### Oil red O staining

Oil red O (Sigma), 0.5 g, and 100 ml isopropanol were mixed, and kept in the dark as stock solution. The working solution was prepared prior to use (6 volumes of stock solution to 4 volumes of distilled water), filtered, and used within 2 hours. The cells fixed with 4% paraformaldehyde for 10 minutes on slides were washed with PBS. RCC frozen tissue sections were stained as well. Oil red O staining was carried out for 8 minutes in the dark. Then, the samples were washed with 60% ethyl alcohol for 5 seconds and counterstained with hematoxylin for 30 seconds. Samples were assessed by microscopy.

### ELISA for FFA quantitation

FFA levels were assessed with FFA ELISA Kit. Tissue samples were obtained from 12 patients treated with sorafenib for the first time and 10 patients resistant to sorafenib. Tissue homogenates were added to plates for 30 minutes at 37°C Then, enzyme and color reagents were added for 10 minutes at 37°C in the dark. Stopping solution was added, and absorbance obtained at 450 nm.

### Electronic microscopy

ACHN, ACHN-R, and ACHN-R + ubenimex (0.25 mg/ml) cells were cultured for 24h, and fixed with 2.5% glutaraldehyde for 1.5 h. After postfixation with 1% osmium tetroxide for 1.5 h, the samples were dehydrated with increasing ethanol concentrations, embedded and sectioned. The resulting sections were stained with uranium acetate and 0.3% lead citrate, and observed on a JEOL-1200EX electron microscope (Jeol, Tokyo, Japan).

### Animal experiments

Five week old female nude BALB/c mice were obtained from VITAL RIVER (Beijing, China). The study protocol was approved by the Animal Ethics Committee of Provincial Hospital Affiliated to Shandong University, and adhered to the Office of Laboratory Animal Welfare requirements. The animal experiments were designed based on preliminary findings. Approximately 5 × 10^6^ ACHN/ACHN-R cells were subcutaneously inoculated into the right flank of mice randomly assigned to 5 groups (5 mice per group). Two weeks later, the mice were treated once daily by gavage with vehicle (DMSO), sorafenib (30 mg/kg), and sorafenib + ubenimex (20 mg/kg), respectively, in both parental and resistance groups. Tumor volumes were estimated every 4 days using the standard formula (volume = length × width ^2^/2). A month later, all mice were sacrificed.

### Statistical analysis

Data were analyzed by Student's *t*-test, with the Statistical Package for Social Science (SPSS for Windows package release 10.0; SPSS Inc., Chicago, IL, USA). *P* < 0.05 was considered statistically significant.

## Abbreviations

RCC: renal cell carcinoma
APN: aminopeptidase N
786-R: sorafenib resistant cell line of 786-O
ACHN-R: sorafenib resistant cell line of ACHN
TKI: tyrosine kinase inhibitor
3MA: 3-Methyladenine
PI3K: phosphoinositide 3-kinase
mTOR: the mammalian target of rapamycin
DMSO: dimethyl sulfoxide
VEGF: vascular endothelial growth factor
VEGFR: Vascular Endothelial Growth Factor Receptor
PDGF: platelet derived growth factor.
